# An Altered Skin and Gut Microbiota Are Involved in the Modulation of Itch in Atopic Dermatitis

**DOI:** 10.3390/cells11233930

**Published:** 2022-12-05

**Authors:** Catharina Sagita Moniaga, Mitsutoshi Tominaga, Kenji Takamori

**Affiliations:** 1 Juntendo Itch Research Center (JIRC), Institute for Environmental and Gender-Specific Medicine, Juntendo University Graduate School of Medicine, Chiba 279-0021, Japan; 2 Department of Dermatology and Venereology, Faculty of Medicine, Tarumanagara University, Jakarta 11440, Indonesia; 3 Anti-Aging Skin Research Laboratory, Juntendo University Graduate School of Medicine, Chiba 279-0021, Japan; 4 Department of Dermatology, Juntendo University Urayasu Hospital, Chiba 279-0021, Japan

**Keywords:** skin, gut, microbiota, itch, atopic dermatitis

## Abstract

Skin and gut microbiota play an important role in the pathogenesis of atopic dermatitis (AD). An alteration of the microbiota diversity modulates the development and course of AD, e.g., decreased microbiome diversity correlates with disease severity, particularly in lesional skin of AD. Itch is a hallmark of AD with unsatisfying treatment until now. Recent evidence suggests a possible role of microbiota in altering itch in AD through gut–skin–brain interactions. The microbial metabolites, proinflammatory cytokines, and impaired immune response lead to a modulation of histamine-independent itch, disruption of epidermal barrier, and central sensitization of itch mechanisms. The positive impact of probiotics in alleviating itch in AD supports this hypothesis, which may lead to novel strategies for managing itchy skin in AD patients. This review summarizes the emerging findings on the correlation between an altered microbiota and gut–skin–brain axis in AD, especially in modulating itchy skin.

## 1. Introduction

Microbiota is the group of microorganisms that inhabit all anatomical sites of the body. Previous studies have been focused on microbiota as an agent of disease; however, recent studies have shown that the microbiota can act as regulators of the immune system in human health [1]. The advanced findings on the potential roles of microbiota in human disease may lead to preventative and/or therapeutic breakthroughs [2].

The imbalance of the microbiota is called dysbiosis, which can cause the development of some inflammatory and systemic autoimmune diseases [2]. The discovery of a widely diverse microbiota leads to the change of the “hygiene hypothesis” into the “biodiversity hypothesis.” The association between the decrease in environmental biodiversity and increase in the prevalence of inflammatory and, especially, allergic diseases has been reported [1].

As the largest organ in human body, the skin is colonized by >100 microbial phylotypes and 1000 different bacterial species, with most of them being harmless or even beneficial to their host [2,3]. Studies have highlighted the diversity and skin site specificity of microbial communities on human subjects [4,5,6], which are highly variable depending on endogenous host factors, topographical location, and exogenous environmental factors [3].

Atopic dermatitis (AD) is a one of the most common chronic inflammatory skin diseases with a complex pathophysiology and various clinical features [7,8]. The pathophysiology of AD consists of complex interactions between epidermal barrier disruption, skin microbiota dysbiosis, and altered type 2 immune responses [8,9]. Chronic itch is known as one of the main symptoms in AD patients especially via non-histaminergic itch signaling pathways, which usually persists despite the administration of medication [10,11,12].

Studies have shown that AD patients exhibit reduced diversity in the skin and gut microbiota, with disease severity being negatively correlated with microbiota diversity in the skin. Moreover, the relationship between the increased level of *Staphylococcus aureus* in the skin, particularly during AD flares, disease severity, and skin microbiota diversity reduction has been reported [13]. Recently, the alterations in the interaction between the microbiota and components of the itch pathway have been arising as an advanced mechanism by which the microbiota are involved in the pathophysiology of AD [13,14,15,16]. However, there have been only a few studies on this issue. Therefore, in this review, we aimed to summarize the updated data on skin and gut microbiota dysbiosis, gut–skin–brain axis, and itch in AD.

## 2. Skin Microbiota

The skin is inhabited by approximately 10^6^ bacteria per square centimeter. The skin microbiota are acquired at birth and undergoes alterations throughout human life. This community stabilizes as we approach adulthood, but it continues to evolve in response to various host and environmental factors that each person might possess, i.e., a personalized skin microbiota [2,17].

Firmicutes, Actinobacteria, Bacteroidetes, and Proteobacteria are prominent in skin microbiota. The most abundant phylum on the skin is the Actinobacteria, whereas Gram-positive *Staphylococcus epidermidis* and *Cutibacterium acnes* are mostly found in the human epithelia and sebaceous follicles, respectively [1,18]. Studies have also demonstrated differences in the skin microbiomes at different life stages [19]. At 2 days of age, babies have area-specific differences in their skin microbiota that might affect the future development of disease [19,20].

The skin microbiota consists of two groups of microbes, e.g., permanent residents and temporary residents (transient microorganisms), which emerge from the environment and persist for hours to days [21]. Skin microbiota and microbial colonization are dependent on the physiology and anatomy of the skin site [17,18]. Further, human skin consists of the following four microenvironments: dry, moist, sebaceous, and others (sweat glands, hair follicles, dermal layers) [4]. Each microenvironment has a distinct microbiota. Corynebacteria, Proteobacteria, and Flavobacteriales are prominent on dry areas, whereas Proteobacteria, Corynebacteria, *Staphylococcus* are prominent on moist areas. Sebaceous microenvironments, such as the face and upper body, are inhabited mainly by *Cutibacterium* and *Staphylococcus* [18].

Each person may have a different skin microbiota. The influencing factors of these differences can be classified into intrinsic and extrinsic factors. Intrinsic factors include age, genotype, body temperature and pH, and host immune system. Extrinsic factors include clothing choices, climate, humidity, antibiotic use, detergent and emollient use, surface contact factors such as antiperspirant, and frequency of hygiene [1].

### 2.1. Role of Skin Microbiota in Atopic Dermatitis

Healthy controls and AD patients showed different configurations of the skin microbiota at different sites. Even though the composition of bacteria was strongly dependent on the anatomic area, the relative abundance of *Staphylococci*, particularly *S. aureus*, was increased across all skin sites in patients with AD, with the largest increases on chronic lesions as compared with acute lesions or non-lesional skin. Up to now, *S. aureus* has been the most studied microbiota in AD patients, followed *by S. epidermidis* and *S. haemolyticus* [22,23].

A low bacterial diversity is the common characteristic of lesional skin in AD [23]. The relative abundance of *S. aureus* is increased, while a decrease in *Cutibacterium*, Proteobacteria, *Streptococcus*, *Corynebacterium*, and *Prevotella* is found [24]. The abundance of *S. aureus* in patients with AD was 70% on lesional skin and 39% on non-lesional or healthy skin of the same patient, confirming the correlation between *S. aureus* and disease severity [25]. The *S. aureus* strain structure isolated from AD lesional skin from that of non-lesional skin is different. Several *S.aureus* proteins are known to adhere to host molecules in the stratum corneum. It has been reported that the severe AD is prone to carry the clonal complex 1 (CC1, better adherence) strains, while asymptomatic individuals carry the CC30 (less adherence) strains [26].

Biofilm formation by AD-associated *S. aureus* has been reported to have a major role in the chronic persistence of *S. aureus* on AD skin lesions and difficulties of eradicating it using antibiotics. The biofilm is the prominent mode of growth of the skin microbiota, which facilitates adhesion and persistence in the cutaneous microenvironment, thus contributing to the skin barrier function and local immune responses [27]. The *S. aureus* biofilm promptly grows on the epidermis, inducing oxygen depletion and disruption in the defensive barrier function of the skin [28].

*S. aureus* activates protease receptors which leads to an alteration of the skin barrier of AD patients by releasing endotoxins and enterotoxins that stimulate mast cells, causing inflammation and impairment of keratinocytes. This activation also increases the production of type 2 cytokines, such as thymic stromal lymphopoietin (TSLP), IL-4, and IL-13 [29]. *S. aureus* secretes proteases and phenol-soluble modulin α (PSMα), which lead to endogenous epidermal proteolysis and skin barrier damage that promoted inflammation in mice. Moreover, clinical isolates of different coagulase-negative staphylococci (CoNS) species residing on normal skin, such as *S. epidermidis, S. hominis,* and *S. lugdunensis*, produced autoinducing peptides that inhibit the *S. aureus* accessory gene regulation system, in turn decreasing PSMα expression. This protective process is impaired when *S. aureus* is the dominant species in the skin [30].

Another well-known factor of AD pathogenesis is the alteration in epidermal lipid metabolism that leads to changes in the composition of fatty acids (FAs), ceramides, and cholesterol [31] and can influence the structure of the microbiota in AD [15]. Baurecht et al. investigated interactions between microbiota structure and diversity and epidermal lipid composition: high levels of unsaturated long-chain FAs were associated with an increase in the abundance of *Cutibacterium* and *Corynebacterium*, while high levels of saturated short-chain FAs were associated with a reduction in the abundance of these genera. Further, an increase in the abundance of *S. aureus* was associated with higher levels of the ceramide CER-[AS] [22]. Li et al. found that the levels of some ceramides (CER[AH]C38–C52, CER[AP]C40), some very-long-chain ceramides (CER[EOH]C66–C70 and CER[EOS]C70), some triglycerides, and unsaturated FAs (16:1 and 18:1) were significantly lower in patients with AD having positive *S. aureus* colonization state than in those without [32]. These studies provide an interesting interaction between epidermal lipid composition and structure and diversity of the microbiota in AD.

### 2.2. Relationship between Skin Microbiota and Itchy Atopic Dermatitis

Recent advances in itch research has elucidated that multiple factors that disturb the normal functional correlation between the skin barrier and microbiota, e.g., protease activity and skin pH, may contribute to the increased activation of the non-histaminergic itch pathway in AD patients. In addition, nerve fibers as itch transmitters may interact with the microbiota and peptides involved in microbial function via an array of receptors [13].

A study by Blicharz et al. evaluated the scoring atopic dermatitis (SCORAD) index components depicting itch intensity (excoriations, subjective evaluation of itch, and sleep loss) in adult AD patients. Lesional and non-lesional skin showed a higher frequency of *S. aureus* colonization than controls (81.8% and 57.6% vs. 5.6%, respectively). *S. aureus* abundance on lesional/non-lesional skin is positively correlated with sleep loss and excoriations. The mean values of excoriations were higher in patients colonized by *S. aureus* than in those without *S. aureus*. This study concluded that *S. aureus* skin colonization may be one of the factors aggravating itch in AD [16].

Commensal and pathogenic species inhabiting the skin both express proteases, which are used as virulence factors. For example, *S. aureus* proteases enable pathogen penetration by degrading collagen and elastin, which are essential components of connective tissue in the dermis, and lead to skin barrier disruption [33]. Proteases cleave the N-terminus of the protease-activated receptors (PAR-2 and PAR-4) that manifest on different cell types, including sensory neurons, and mediate itch [34,35]. One of the characteristics of AD is proteases overactivity. Comparative analysis of the complete transcriptome from the skin biopsies from healthy controls and AD patients showed that the genes encoding PAR-2 and -4 (F2RL1 and F2RL3) were higher in itchy AD skin than in skin from healthy controls Likewise, the largest increase in PAR-2 expression occurred in itchy AD skin, followed by less increase in non-lesional skin [36].

*S. aureus*, such as δ-toxin, can directly generate type 2 immune response by activating the immune cells and producing inflammatory mediators, such as IL-4, IL-13, and TSLP [14,27]. Oetjen et al. reported that the dorsal root ganglion in mice and humans expressed IL-4Rα and IL-13Rα, and that IL-4 and IL-13 can directly activate sensory neurons, thereby inducing an itch [37]. Further, IL-4 and IL-13 can reduce the expression of anti-microbial peptide (AMP), which causes a more permissive-skin AD to *S. aureus* colonization [27].

The structure and function of the microbiota are also significantly influenced by the skin pH and, thus, also by itch. Commensal bacteria thrive on healthy skin with an acidic pH (pH 4–6), while pathogenic bacteria such as *S. aureus* thrive at neutral pHs. AD is associated with an increase in skin pH to ≥6.5, promoting the colonization of *S. aureus*, directing to dysbiosis and an increased abundance of microbiota-derived serine proteases [38]. Moreover, the enhancement of pH also activates endogenous skin proteases (pH optimum at 8), leading in further activation of the keratinocyte PAR-2 receptors and non-histaminergic pathway [39].

Delta-hemolysin and PSMα produced by *S. aureus* induced degranulation of human mast cells in a dose-dependent manner and mediates skin pathology in AD [40,41]. The antigens of *S. aureus* have been reported to induce IL-31, which is the first cytokine that is known to mediate itch by directly operating on sensory neurons [42], in vivo and in vitro in individuals with AD, implying that *S. aureus* can aggravate not only disease flares but also the itch in AD [42]. Whether this is secondary to the hyperstimulation of the Th2-type response or other mechanisms is not certain [16].

The abovementioned data revealed that *S. aureus* colonization of the skin lesions and non-lesional skin is associated with higher values of itch determinants in AD. *S. aureus* seems to cause hyperstimulation of the immune system and overexpression of itch mediators, suggesting that the overabundance of *S. aureus* may contribute to the intensity of pruritus through a non-histaminergic mechanism [16].

### 2.3. Neuropeptides, Skin Microbiota, and Itch

Several neuropeptides secreted by the skin, including substance P (SP), are known to be involved in the pathophysiology of itch in AD. The increased virulence of some bacteria, including *Bacillus* and *Staphylococci*, can be induced by localized increases of SP in the skin. In addition, the calcitonin gene-related peptide has been reported to modulate the virulence of *S. epidermis*, another key component of the skin microbiota. Thus, neuropeptides in the skin appear to act as host signals for the microbiota and play an important role in skin homeostasis and disease pathophysiology [43].

Thirteen mammalian Toll-Like Receptors (TLRs) have been identified and characterized. TLR3, TLR7, TLR8, and TLR9 are localized in the endosome to detect nucleic acids derived from viruses, bacteria, or damaged cells. TLR3 recognizes viral double-stranded RNA (dsRNA), TLR7 and TLR8 recognize single-stranded RNA (ssRNA) found during viral replication [44]. Studies have suggested that sensory neurons can detect the microbiota via TLR. Liu et al. reported that functional TLR7 is expressed in C-fiber primary sensory neurons that highly co-localized with transient receptor potential subtype V1 (TRPV1) and gastrin-releasing peptide, and it is an important mediator of itch transmission [45]. TLR3 is also displayed by small-sized primary sensory neurons in dorsal root ganglions, and its binding with ligands stimulates neuronal activity and itch [46].

### 2.4. Skin–Brain Axis and Itchy Skin

It is known that stress exacerbates itch via the skin–brain axis. Recently, the microbiota has emerged as a major player to regulate this axis, especially during stress aroused by actual or perceived homeostatic challenge. The routes of communication between the microbiota and brain are gradually being resolved and involve neurochemicals (i.e., acetylcholine, histamine, catecholamines, corticotropin) that originate from the microbiota [15].

Since stress can aggravate itch, it is believed that the brain is occupied in the final common stage of itch processing [47]. Stress acts via the central nervous system (CNS) and changes the microbiota through the release of neurochemicals [48]. The component of the stress response, such as glucocorticoids, may repress AMP release/localization in the epidermis, impair skin barrier, and promote host susceptibility to infection. Skin microbiota, especially the CoNS, are sensitive to catecholamines. Catecholamines also strengthen bacterial attachment to host tissues and increase bacteria virulence. Catecholamines induce the biofilm formation of *Pseudomonas aeruginosa* and *Escherichia coli*, which enhances USA300 methicillin-resistant *S. aureus* virulence. Norepinephrine, epinephrine, dopamine, and their structurally related inotropes (dobutamine and isoprenaline) raise staphylococcal growth by 5-log orders or more. [15]. Thus, the effect of stress on the skin microbiota may be twofold: dampening the host defense to infection and adjusting the microenvironment ideal for pathogens [49].

The amygdala is thought to be vulnerable to microbial influences [50]. Study of germ-free (GF) mice suggested that the amygdala transcriptome becomes hyperactive in the absence of microbiota [51]. This hyperactive state is in line with the altered pain sensitivity [52] and stress response in GF mice [53]. However, how microbial signals navigate through the skin–brain axis to reach the amygdala specifically is still unknown.

### 2.5. Association of Skin Microbiota and Itchy Skin of Non-AD Disease

Cirrhosis is a late stage of scarring (fibrosis) of the liver. Patients with cirrhosis have intestinal dysbiosis and are prone to itching. Bajaj et al. reported that the composition of microbiota at all skin sites differed between controls and patients with cirrhosis and between patients with compensated and decompensated cirrhosis. Skin microbiomes of patients with cirrhosis (especially those with decompensation) contained a higher relative abundance of Gammaproteobacteria, Streptococaceae, and Staphylococcaceae. These bacterial taxa were associated with serum levels of autotaxin and bile acids (BAs), which were higher in patients with VAS scores ≥ 5 [54].

Senile pruritus is a common skin disease in the elderly. A study among senile pruritus patients showed that, compared with the healthy control group, the patients had significantly lower skin hydration and higher pH value [55]. The α-diversity of skin microbials was significantly increased in senile pruritus patients, with an increase of *Acinetobacter* and *Lactobacillus*, and decrease of *Cutibacterium*. The pH value and skin hydration were positively associated with observed species diversity and *Lactobacillus*, whereas the transdermal water loss was negatively related to *Lactobacillus*. Thus, the damaged skin barrier function and skin dysbiosis complemented each other and were probably related with the occurrence of senile pruritus; however, the definite mechanism needs further study [55].

## 3. Gut Microbiota

The human gut possesses one of the most complex and generous ecosystems colonized by more than 100 trillion microorganisms [56,57]. The predominance of the bacteria residing in the adult gut belong to two bacterial phyla, the gram-negative Bacteroidetes and the gram-positive Firmicutes; and the others represented at subdominant levels are the Actinobacteria, Fusobacteria, and Verrucomicrobia phyla, which vary dramatically among individuals [58,59,60]. The concept of ‘enterotype clusters’ was introduced to effectively classify the different microbial colonizations and structures [58,59]. The results of metagenomics sequencing of fecal samples confirmed the three robust clusters dominated by *Bacteroides* (enterotype 1), *Prevotella* (enterotype 2), and *Ruminococcus* (enterotype 3) [3].

The composition and functions of the gut microbiota are affected by several factors including genetics, mode of delivery, age, diet, geographic location, and medical treatments [61,62]. The gut is an anaerobic environment in which native species have co-evolved with the host. While the aerobic pathogenic species cannot invade and colonize this environment, diseases may develop when anaerobic and facultative pathogenic species invade the gut. High diversity defines healthy human gut microbiotas, while reduction in diversity may be associated with dysbiosis [59]. Studies have shown a possible direct relationship between gut dysbiosis and a variety of diseases (the gut–brain axis), such as inflammatory bowel diseases including colitis and Crohn’s disease, obesity, cancer [61,62], Parkinson’s disease, Alzheimer’s disease, multiple sclerosis [63], and neurophyschiatric disorders [64].

Starting at the first month of life, the gut microbial community plays an essential role in the maturation of the human immune system. The acquisition of the gut microbiota begins at birth, after which it diversifies at approximately 6 months after birth. Through the development process, the stability of the gut microbiota is achieved during the third year of life [65]. Gut microbiotas produce many metabolites and signal molecules, such as post-translationally modified peptide, amino acid metabolites, acids (short-chain), oligosaccharides, glycolipids, and non-ribosomal peptides, which affect the systemic immune response of an individual [14,66]. Therefore, the gut microbiota are a potential target for regulating immune responses in the host [67].

### 3.1. Atopic Dermatitis and Gut Microbiota

The gut dysbiosis association in patients with AD has been introduced for some time [68]. A reduction of diversity of the gut microbiota has also been reported to cause AD development [69]. A cross-sectional study among children showed that a diversity of gut microbiota was closely associated with a decreased risk of AD [70]. It is believed that the gut microbiota in early life is associated with age of onset, severity, flares, remission, and phenotype of AD. In addition to the gut microbiota diversity in itself, the interactions between specific gut microbiota, established immune systems, and harmonization of the gut microbiota and the host may define the development of AD [71].

The gut microbiota in infants with AD are significantly less diverse than those in healthy controls, and several signature microbes from the genera *Lactobacillus, Bacteroides*, and *Clostridium* have been identified [72]. Higher levels of *Clostridium difficile*, *E. coli*, and *Bacteroides* were found in the gut of atopic infants compared to healthy controls [14,69,73,74]. *Clostridium* and *E. coli* in the gut can participate in the occurrence of inflammatory conditions [73]. Contrarily, lower levels of *Akkermansia, Lactobacillus, Faecalibacterium prausnitzii,* and *Bifidobacterium* were found in AD patients, compared to healthy controls [67,74,75,76]. Butyrate-producing bacteria are more abundant in healthy infants or infants with mild AD, as compared to infants with severe AD [77].

The gut microbiota can control the immune system of the body and skin. The condition of the gut microbiota greatly affects the differentiation of naïve T cells to other types of Th cells, such as Th1, Th2, Th17, or Foxp3^+^ Tregs. Tregs prevent dysfunctional T cells from differentiating into Th cells and inhibit the inflammatory activities of immune cells [65,78]. *Bifidobacterium*, *Lactobacillus*, *Clostridium*, *Bacteroides*, *Streptococcus*, and their metabolites, such as propionic and butyric acids, are known for their capability in inducing the polarization and expansion of Treg cells [79].

The gut flora produces a vast amount of metabolites, which may enter the circulation, traveling throughout the body and affecting distant sites of the organism. This process can reach high levels when the epithelial barrier integrity of the gut is disrupted, leading to increased intestinal permeability, referred to as the “leaky gut syndrome” [80]. The short-chain fatty acids (SCFAs), such as butyrate, propionate, acetate, and lactate, are products of fiber fermentation by the gut microbiota, which are known to promote epithelial barrier integrity of the gut and exert anti-inflammatory effects [81]. Intriguingly, intestinal dysbiosis has been demonstrated in AD patients, and a clear reduction of SCFAs has been detected. Hence, a leaky gut in AD patients favors toxins, poorly digested foods, and gut microorganisms to penetrate the body circulation and leads to skin inflammation. When these reach the skin, a strong Th2 reaction may be induced, causing further tissue damage [82].

The “gut–skin” axis has been proposed and is recognized as a new target to prevent and treat AD. Gut and skin have several similar characteristics and are parts of the overall immune and endocrine systems. The development of gut diseases is commonly accompanied by cutaneous lesioned manifestations, implying that the association between them may affect each other’s states [67]. Especially, the colonization and alteration of the gut microbial were reported prior to any clinical manifestations in early life, indicating gut microbial symbiosis as one of the causes of AD [83]. Advanced studies support the presence of a gut–skin axis that is mediated by neuroendocrine molecules produced by the gut microbiome [84,85]. The data suggest that compositional and proportional differences in the gut microbiome are connected to the generation of diverse favorable neurotransmitters and neuromodulators, which are associated with the degree of AD symptoms [71]. Therefore, targeting gut microbial alterations may be an alternative to regulate immune responses and ameliorate cutaneous health in AD patients [67].

### 3.2. Gut Microbiota and Itch in AD

As explained previously, it is known that the gut microbiota and their dependent metabolites are associated with the development of chronic diseases, such as AD, which is characterized by chronic itch. Li et al. investigated the gut microbiota environment in diphenylcyclopropenone (DCP)-evoked chronic itch model in mice. This study showed the microbiota composition in mice was significantly altered by the DCP-evoked chronic itch. DCP mice showed significant decrease in α-diversity value and relative abundance of 48 bacteria at six phylogenetic levels (phylum, class, order, etc.) compared with control mice. Contrarily, the relative abundance of four bacteria at the genus, phylum, and species level increased in the DCP mice compared with the control mice. These results suggest that the gut microbiota may play an important role in DCP-evoked chronic itch in mice [86].

The microbiota of the gut and skin affect each other through neuroendocrine signaling. This effect can occur via two routes, i.e., direct and indirect [87]. An example of direct signaling is tryptophan produced by intestinal microbes causing skin itching in AD patients. On the contrary, γ-aminobutyric acid produced by *Lactobacillus* and *Bifidobacterium* in the gut suppresses itch of the skin [84,87]. Through indirect channels, intestinal microbes adjust the concentration of IL-10 and IFN-γ in the bloodstream, which can lead to abnormal changes in brain function, resulting in stress. Cortisol, a representative stress hormone in humans, can alter the gut epithelium permeability and barrier function by changing the composition of the gut microbiota [14].

**Figure 1:**
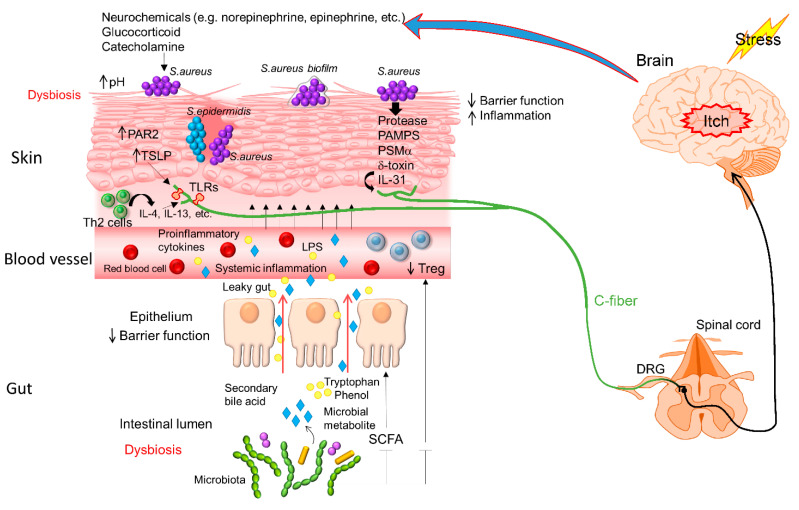
Proposed mechanism of a microbiota dysbiosis modulates itch in atopic dermatitis. The leaky gut condition due to gut dysbiosis enables microbiota metabolites and proinflammatory cytokines to circulate and reach the skin, e.g., tryptophan, LPS, and others. In the skin, decreased microbial diversity and *S. aureus* colonization lead to disruption of skin barrier function and the production of pruritoceptors, such as IL-4, IL-13, TSLP, IL-31, and so on, which trigger an itch sensation in AD.

Cirrhosis is largely an acquired disorder with multi-system consequences. As mentioned previously, cirrhosis patients have intestinal dysbiosis and are prone to itching. In addition to the alteration of the skin microbiota, this study found significant differences in the stool and salivary microbiota in those with greater itching, which were different from the taxa that separated controls from cirrhosis or compensated from decompensated cirrhosis. Bajat et al. also identified alterations in the fecal microbiome of patients with cirrhosis, i.e., fecal microbiota of patients with cirrhosis had a higher relative abundance of Gammaproteobacteria than controls, which are associated with itching intensity and itch modulators. This implies that a specific signature of the microbial composition that is present in the gut could be associated with skin itching [54]. A summary of components involved and the proposed mechanism of an altered microbiota in the skin and gut may modulate itch in AD as explained in Table 1 and Figure 1.

## 4. Different Body Sites Have Different Microbiota with Different Effects on AD

The composition, diversity, and interactions with the host of the microbial communities in different body habitats are site specific, with alterations in the diversity or metabolic activity of these unique microbiomes resulting in inflammation and atopic disease [88,89]. In a study comparing the structure and predicted functions of microbial communities from the skin, oral cavity, and gut, Li et al. [90] revealed that healthy controls showed different alterations in community-level diversity between microbiomes from the same body habitats (α-diversity) and patients with AD. In AD patients, the most significant alleviation in α-diversity was seen in the skin, followed by the oral cavity, while no differences in community structure were found in the gut microbiota. In addition, alterations in the level of enrichment of specific microbes at the different body habitats were observed in patients with AD as compared with controls. *Staphylococcus* was enriched in the skin samples from patients with AD, while *Halomonas* was more generous in the skin of healthy controls. Interestingly, *Halomonas* was enriched in the oral cavity of AD patients as compared with in the controls, suggesting that the patterns of enrichment in AD vary according to body habitat. The clinical severity of AD also differentially correlated with levels of diversity in the different regions: in the skin, microbiome α-diversity was negatively correlated with skin disease severity, while a positive correlation was seen in the oral cavity. Analysis of the predicted functional profiles of the microbiome from each habitat sampled revealed an inverse connection between pathway enrichment in the skin and oral microbiomes: the tryptophan metabolism was enriched in the oral cavity of patients with AD but was attenuated in the skin. In summary, this study showed that the different body regions in AD patients possessed differential changes in the structure of microbial communities, with lower levels of diversity both within and between the skin and oral microbiomes, simultaneously with inverse associations in predicted microbial function between the two microbial communities [90].

### Microbiome-Based Treatments for Itchy AD

Looking at the great role of microbiota on AD, we can hypothesize that microbiota-based therapies are needed to restore a healthy skin and gut microbiota in AD patients. Some therapies include probiotics treatment, repopulating AD lesions with beneficial commensals, phage therapies, small molecules and peptides that counteract *S. aureus* colonization, humanized monoclonal antibodies that target bacterial toxins, quorum sensing inhibitors that block virulence factors, bacteriotherapy, and the excimer light treatment [91,92,93,94].

Probiotics by definition are ‘live microorganisms, which, when consumed in adequate amounts, confer a health effect on the host’ [95]. Prebiotics are non-digestible ingredients that beneficially affect the host by selectively stimulating growth or limiting gut microbiota [96]. Probiotics and/or prebiotics, as the common regulator for gut microbiota, have been used to alleviate AD clinical symptoms, but with controversial outcomes (positive or ineffective). This is related to the complex interaction between the immune response, gut microbiota, and metabolic activity in the host [67]. Actual health effects have been reported for specific strains of the following genera: anaerobic microbes (*Lactobacillus*, *Bifidobacterium*, *Saccharomyces*, *Enterococcus*, *Streptococcus*, *Pediococcus*, *Leuconostoc*, *E. coli*), and the aerobic Bacillus strains [95]. Postbiotics are metabolites from intestinal bacteria, which include microbial cells, cell constituents, and metabolites [14,97]. Moroi et al. showed that the skin severity scores were significantly improved after treatment with postbiotic *L. paracasei* K71 in adult patients with AD compared to that after placebo administration, whereas the itch scores did not [98]. One of the common problems among patients with chronic kidney disease is pruritus, which was associated with the accumulation of calcium phosphate (CaP) under the skin. Skin *C. acnes* can solubilize CaP by the production of SCFAs, such as the N-[2-(2-Butyrylamino-ethoxy)-ethyl]-butyramide (BA-NH-NH-BA), a butyric acid derivative. Topical application of BA-NH-NH-BA onto mouse skin efficaciously improved CaP-induced skin itching [99]. Furthermore, it is suggested that postbiotic compounds from *Lactobacilli* spp. can employ immunomodulation activity by increasing the levels of Th1-associated cytokines and reducing Th2-associated cytokine levels [97]. Table 2 shows the microbiome-based treatment studies related to itching in AD.

Most probiotics reduced the SCORAD scores and even decreased the risk of developing AD. Niccoli et al. evaluated the clinical efficacy of a lyophilized form of *Lactobacillus salivarious* LS01 in pediatric AD patients. The results of this study reported a significant decrease in SCORAD value and significant improvement in itch intensity when compared to the placebo control group. These reductions persisted after treatment suspension [100]. Further, Kim et al. investigated the attenuation of the AD symptoms using *L. paracasei* KBL382 isolated from the feces of healthy Koreans. Oral administration of *L. paracasei* KBL382 for 4 weeks significantly reduced AD-associated skin lesions, number of scratches, TSLP and IL-31 levels in the skin, and immune cell infiltration in *Dermatophagoides farinae* extract-induced AD in mice. In addition, the administration of KBL382 dramatically changed the composition of the gut microbiota in AD mice. Therefore, it is hypothesized that the administration of KBL382 significantly ameliorates AD-like symptoms by regulating the immune response and altering the composition of the gut microbiota [101].

Many studies reported that oral probiotic formulations consisted of *Lactobacillus* strains; however, only several studies have investigated the positive effects of other bacterial strains or mixtures of different probiotic bacterial strains in the management of AD. Matsumoto et al. reported that the administration of *Bifidobacterium animalis* subsp. *lactis* LKM512 alleviated itch and considerably improved the dermatology-specific quality-of-life scores when compared with the controls. The antipruritic and antinociceptive metabolite kynurenic acid production was increased after administration of LKM512, suggesting an antipruritic effect of *B. animalis* [102]. Kim et al. investigated the immunomodulatory effects of oral yeast-extracted β-1,3/1,6-glucan and/or *Lactobacillus plantarum* LM1004 against AD-like symptoms in AD-induced animal models of rat (histamine-induced vasodilation) and mouse (pruritus and contact dermatitis). The treatment group showed significantly reduced vasodilation in the rat model, and pruritus, edema, and serum histamine levels in the mouse models. Moreover, in rats with no AD induction, the same treatments significantly increased the relative abundance of the phylum Bacteroidetes and the genus *Bacteroides*. In the treated groups, Bacterial taxa associated with butyrate production, such as Lachnospiraceae and Ruminococcaceae at the family level and *Roseburia* at the genus level, were increased. These findings suggest that the dietary supplementation of β-1,3/1,6-glucan and/or *L. plantarum* LM1004 has an immense potency for the treatment of AD via mechanisms that might involve modulation of host immune systems and gut microbiota [103].

Phenols, for example, phenol and p-cresol produced by gut bacteria, are regarded as bioactive toxins and serum biomarkers of a disturbed gut environment, which can accumulate in the skin via the circulation and disrupt keratinocyte differentiation in hairless mice. It has been reported that daily intake of the probiotic *Bifidobacterium breve* together with the prebiotic galactooligosaccharide reduced serum total phenol levels and improved skin hydration in healthy adult women [104]. Restoring a diverse microbiota that includes commensals will improve the community’s resistance to colonization with pathobionts. *S. epidermidis* application on healthy individuals increased the skin lipid content and lowered skin acidic conditions [105]. Moreover, a clinical study demonstrated that oral supplementation with a probiotic *L. paracasei* NCC 2461 decreased skin sensitivity and increased barrier function in a capsaicin-treated group [106]. A compromised skin barrier is known as a very important factor in treating itch, suggesting the importance of oral probiotics in the treatment of itch in AD.

Nakatsuji et al. demonstrated that the topical application of commensal skin bacteria (autologous microbiome transplant, *S. hominis*, and *S. epidermidis*) is effective in protecting against pathogen species, with reduced *S. aureus* colonization and inhibition of PSMα, improvement of clinical symptoms (including itch), and decreased local inflammation [107,108]. Another study also conducted a first-in-human topical microbiome transplantation after collecting the commensal *Roseomonas* mucosa from healthy subjects. A significant decrease in SCORAD score and pruritus was noted after the treatment, leading to a lesser need for topical steroids [109].

## 5. Conclusions

Several studies have demonstrated the link between skin and gut dysbiosis and skin homeostasis imbalances in the pathophysiology of AD. Recently, the interplay between this concept and itch as a distinctive hallmark of AD has also been investigated. Studies have shown that the alteration of microbiota in the skin and gut (attenuation of microbiota diversity) interferes with the mechanism of itch in AD through a non-histaminergic pathway, perturbation of skin barrier, and central sensitization. Moreover, microbiota-derived treatment, especially probiotics, has proven its benefit in relieving itch in AD patients. Prevention and therapy of microbiome dysbiosis could help alleviate itch related to AD. Further insights into the interactions between human microbiota and itch will provide new targets for future treatment of itchy skin in AD patients.

## Figures and Tables

**Table 1:** The components of skin and gut alterations in the modulation of itch in atopic dermatitis.

	Skin	Gut
Alteration of microbiota	Low skin microbiota diversity - Decreased Cutibacterium, Proteobacteria, Streptococcus, Corynebacterium, Prevotella - Increased S. aureus (primary), S. epidermidis, S. haemolyticus	Low gut microbiota diversity - Decreased Lactobacillus, Bifidobacterium, Akkermansia, Corynebacterium, F. prausnitzii - Increased Escherichia coli, Clostridium difficile, Bacteroides
Metabolites	*S.aureus*-derived toxins, e.g., proteases, PSMα, δ-toxin, delta-hemolysin; neuropeptides (e.g., substance P, calcitonin gene-related peptide), TLRs; neurochemicals during stress induced itch	Decreased SCFAs (especially butyrate), Tregs; increased tryptophan
Proposed mechanism	Activation of PARs and production of type 2 cytokines by δ-toxin → sensory neurons; increased of skin pH; delta-hemolysin-induced IL-31 production; TLRs activate sensory neuron	leaky gut syndrome

Note: PSMα = phenol-soluble modulin α; SCFs = short-chain fatty acids; TLRs = toll-like receptors.

**Table 2:** Microbiome-based treatments for AD itch.

No.	Microbiota Treatment	Component	Subject	Mode of Administration	Alteration of Microbiota	Result	Reference
1.	Probiotic	*Lactobacillus salivarious* LS01	Pediatric AD	Oral	NA	Improvement of itch and SCORAD index	[100]
2.	Probiotic	*Lactobacillus paracasei* KBL382	*Dermatophagoides farinae* extract-induced AD mice model	Oral	Gut (from cecum, human)	Improvement of number of scratches, AD-like skin lesion, Foxp3^+^; reduction of IL-31; altered gut microbiota and metabolites	[101]
3.	Probiotic	*Bifidobacterium animalis subsp. lactis* LKM512	Adult AD	Oral	NA	Improvement of itch and dermatology-specific quality-of-life scores	[102]
4.	Synbiotic	Yeast-extracted β-1,3/1,6-glucan and/or *Lactobacillus plantarum* LM1004	AD-induced animal models of rat (histamine-induced vasodilation) and mouse (pruritus and contact dermatitis)	Oral	Gut (from fecal, rat)	Reduction of vasodilation (rat model), and pruritus, edema, and serum histamine (mouse model); altered gut microbiota	[103]
5.	Synbiotic	*Bifidobacterium breve* and galactooligosaccharide	Healthy adult	Oral	NA	Improvement of skin hydration	[104]
6.	Autologous microbiome transplant	*S. epidermidis*	Healthy adult	Topical	NA	Improvement of skin lipid; reduction of water evaporation and skin pH	[105]
7.	Probiotic	*L. paracasei* NCC 2461	A capsaicin test in healthy adult	Oral	Gut (from fecal, human)	Decreased skin sensitivity; increased rate of barrier function recovery	[106]
8.	Autologous microbiome transplant	*S.hominis* and *S. epidermidis*	Adult AD	Topical	Skin (from skin, human)	Supression of *S. aureus* colonization	[107,108]
9.	Beneficial commensals	*Roseomonas mucosa*	Adult and pediatric AD	Topical	Skin (from skin, human)	Improvement of subjective regional itch, total itch score, and SCORAD score	[109]

Note: AD = atopic dermatitis; SCORAD = Scoring Atopic Dermatits.
